# Low incidence of delirium in patients followed by physiotherapists in the ICU

**DOI:** 10.1186/cc12619

**Published:** 2013-06-19

**Authors:** KT Timenetsky, D Carnieli-Cazati, CS Azevedo, RC Eid

**Affiliations:** 1 Hospital Israelita Albert Einstein, Morumbi, Sao Paulo, SP, Brazil

## Introduction

Delirium is an acute temporary and fluctuating mental-organic syndrome, characterized by a global impairment of cognitive function, reduced level of consciousness, attentional deficits and altered sleep-wake cycle, and changes in arousal (hyperactive, hypoactive, or mixed). The Confusion Assessment Method (CAM and CAM ICU) [1] is a diagnostic assessment instrument for delirium and can lead physiotherapeutic treatment, aiming to optimize patient's recovery, reinforce the importance of preventive and therapeutic measures, and appraise the multidisciplinary treatment approach in this severe complicating syndrome. Delirium is present in 20 to 40% of ICU patients. So far we have no data regarding the incidence of delirium at the ICU in patients followed by physiotherapists. The objective of this study was to verify the incidence of delirium through the CAM ICU instrument in ICU patients followed by physiotherapists.

## Methods

Trained and capacitated physiotherapists applied the CAM ICU in patients admitted to Albert Einstein Jewish Hospital ICU, older than 18 years old and with 24 hours physiotherapy assistance per day. The content and level of consciousness investigation was performed daily in all physiotherapy sessions, which allowed characterizing the need to apply the CAM ICU. These data were evaluated twice a week during a 30-day period through medical charts.

## Results

During the study period, 226 patients were evaluated by physiotherapists, median age 70 years old (range 21 to 92), and 60% were male. The clinical admission diagnoses were: 36% sepsis, 20% cardiac, 19% neurologic, 11% respiratory, 4% orthopedic, 3% liver and gastric patients, and 2% vascular. The mean Simplified Acute Physiology Score (SAPS 3) was 45 points. We found a delirium incidence of 7% (*n = *16), of these patients 25% were under mechanical ventilation and 25% were spontaneously breathing. In regards to the clinical admission diagnosis, delirium was present in 20% vascular patients, 16% neurologic patients, 14% liver patients, 8% respiratory patients and 2% cardiac patients. We found no correlation of delirium with clinical admission diagnosis (*P *= 0.76; *r *= -0.054).

## Conclusion

There was a low incidence of delirium in ICU patients followed by physiotherapists. This may be due to physiotherapy and multi-professional team interventions performed earlier.

## References

[B1] Bergeron N Skrobik Y Dubois MJ Delirium in critically ill patients Crit Care 2002 17 181 182 10.1186/cc1482 12133171 PMC137438

